# Ring opening metathesis polymerization-derived block copolymers bearing chelating ligands: synthesis, metal immobilization and use in hydroformylation under micellar conditions

**DOI:** 10.3762/bjoc.6.28

**Published:** 2010-03-23

**Authors:** Gajanan M Pawar, Jochen Weckesser, Siegfried Blechert, Michael R Buchmeiser

**Affiliations:** 1 Lehrstuhl für Makromolekulare Stoffe und Faserchemie, Institut für Polymerchemie, Universität Stuttgart, Pfaffenwaldring 55, D-70550 Stuttgart, Germany; Tel.: +49 (0)711-685-64075; Fax: +49 (0)711-685-64050; 2 Institut für Chemie, Technische Universität Berlin, Straße des 17. Juni 135, D-10623 Berlin, Germany

**Keywords:** block copolymers, catalysis, hydrophilic polymers, metathesis, micelles

## Abstract

Norborn-5-ene-(*N,N*-dipyrid-2-yl)carbamide (**M1**) was copolymerized with *exo,exo*-[2-(3-ethoxycarbonyl-7-oxabicyclo[2.2.1]hept-5-en-2-carbonyloxy)ethyl]trimethylammonium iodide (**M2**) using the Schrock catalyst Mo(*N*-2,6-Me_2_-C_6_H_3_)(CHCMe_2_Ph)(OCMe(CF_3_)_2_)_2_ **[Mo]** to yield poly(**M1**-*b*-**M2**). In water, poly(**M1**-*b*-**M2**) forms micelles with a critical micelle-forming concentration (cmc) of 2.8 × 10^−6^ mol L^−1^; Reaction of poly(**M1**-*b*-**M2**) with [Rh(COD)Cl]_2_ (COD = cycloocta-1,5-diene) yields the Rh(I)-loaded block copolymer poly(**M1**-*b*-**M2**)**-Rh** containing 18 mg of Rh(I)/g of block copolymer with a cmc of 2.2 × 10^−6^ mol L^−1^. The Rh-loaded polymer was used for the hydroformylation of 1-octene under micellar conditions. The data obtained were compared to those obtained with a monomeric analogue, i.e. CH_3_CON(Py)_2_RhCl(COD) (**C1**, Py = 2-pyridyl). Using the polymer-supported catalyst under micellar conditions, a significant increase in selectivity, i.e. an increase in the *n*:*iso* ratio was accomplished, which could be further enhanced by the addition of excess ligand, e.g., triphenylphosphite. Special features of the micellar catalytic set up are discussed.

## Introduction

Catalysts bound to amphiphilic block copolymers find increasing use in micellar catalysis since they combine the advantages of both homogeneous and heterogeneous catalysis in one system. Thus, with catalysts permanently linked to the block copolymer, metal leaching is substantially reduced and allows for the separation/reuse of the catalyst [1–7]. In cases where reactions are run in polar media, the catalyst is best located inside the hydrophobic micellar core, where, upon micelle formation of the functionalized block copolymer, the monomer will also accumulate. This leads to high educt concentrations at the polymer-bound catalyst, often resulting in high reaction rates in water [8]. We recently reported on the synthesis of Rh^I^ and Ir^I^ complexes of *N,N*-dipyrid-2-ylacetamide and their use in hydroformylation reactions [9]. Here, we report on the immobilization of a Rh-*N,N*-dipyrid-2-ylacetamide-based catalyst on a soluble, amphiphilic, ring-opening metathesis polymerization- (ROMP) derived block copolymer and its use in hydroformylation [10] under micellar conditions [3,5,11]. This medium activity and selectivity dipyrid-2-ylamide-based Rh(I)-catalyst was chosen in order to identify the potential advantages of a micellar setup.

## Results and Discussion

### Synthesis of monomers

**Scheme 1:**
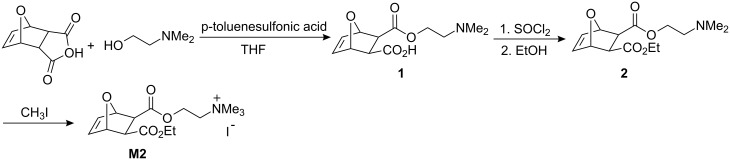
Synthesis of **M2**.

The synthesis of norborn-5-ene-(*N,N*-dipyrid-2-yl)carbamide (**M1**) was accomplished via reaction of norborn-5-ene-2-ylcarboxylic acid chloride with dipyrid-2-ylamine in the presence of triethylamine as described elsewhere [9]. *exo,exo*-[2-(3-Ethoxycarbonyl-7-oxabicyclo[2.2.1]hept-5-ene-2-carbonyloxy)ethyl]trimethylammonium iodide (**M2**) was prepared in a four-step procedure (Scheme 1). It entailed the reaction of 7-oxanorborn-5-ene-2,3-dicarboxylic anhydride with 2-(*N,N*-dimethylamino)ethan-1-ol, conversion of the free carboxylic acid **1** into the corresponding acid chloride with SOCl_2_ and reaction of the acid chloride with dry ethanol to form the diester **2**. Finally, compound **2** was converted into **M2** via quaternization of the tertiary amine with methyl iodide.

### Synthesis of homo- and block copolymers via ROMP [12–13]

ROMP has already been used for the synthesis of micelle-forming block copolymers [14], however, the one used in this study, i.e. poly(**M1**-*b*-**M2**), required special attention. Though **M1** contains a chelating ligand and can be polymerized by both Schrock and Grubbs-type initiators [15–18]. **M2** is particularly problematic since it contains a quaternary ammonium moiety and has the ability to alkylate the phosphane or pyridine ligands of 1^st^-, 2^nd^-, and 3^rd^-generation Grubbs-type initiators. We therefore chose one of the most active Schrock-type initiator [19–21] for polymerization, i.e. Mo(N-2,6-Me_2_-C_6_H_3_)[CHC(CH_3_)_2_Ph][OCMe(CF_3_)_2_]_2_ (**[Mo]**) [22].

**Scheme 2:**
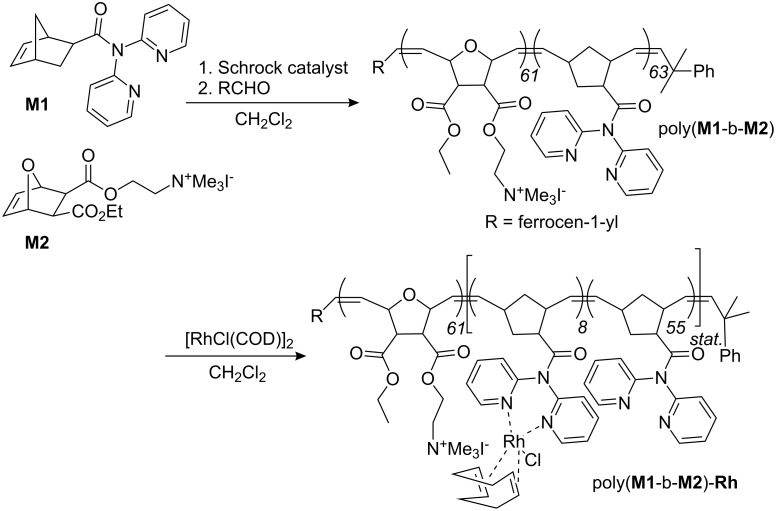
Synthesis of poly(**M1**-*b*-**M2**) and of the micellar catalyst poly(**M1**-*b*-**M2**)-**Rh**.

Both the homopolymerization of **M1** and **M2** by the action of **[Mo]** proceeded smoothly at room temperature. Conversion of monomer **M1** reached 100% after 10 min (Figure S1, Supporting Information File 1). The corresponding block copolymer poly(**M1**-*b*-**M2**) (*M*_n_ = 44100 g mol^−1^, PDI = 1.38, Scheme 2) was prepared in 76% isolated yield by starting the polymerization with **M1** and adding **M2** after 20 min. The GPC-traces of the first and second block are shown in Figure S2 (Supporting Information File 1) and indicate that the diblock copolymer formed quantitatively. From GPC analysis, degrees of polymerization of 63 and 61 were found for the poly(**M1**) and the poly(**M2**) block, respectively.

### Critical micelle forming concentrations, metal loading of poly(**M1**-*co*-**M2**)

The cmc of poly(**M1**-*co*-**M2**) was determined by fluorescence spectroscopy [23]. For measurements in water, 6-*p*-toluidene-2-naphthylsulfonic acid (10^−6^ mol/L) was used as the fluorescence probe. A cmc_water_ for poly(**M1**-*b*-**M2**) of 2.8 × 10^−6^ mol L^−1^ was determined (Figure S3, Supporting Information File 1). Next, poly(**M1**-*b*-**M2**) was loaded with Rh(I) via reaction of poly(**M1**-*b*-**M2**) with [RhCl(COD)]_2_ (COD = cycloocta-1,5-diene) to yield poly(**M1**-*b*-**M2**)**-Rh** (Scheme 2). A metal loading of 18 mg Rh/g polymer was achieved, which corresponds to 12% of the theoretical loading (assuming the formation of a mono-dipyridyl-Rh(I) complex as observed for **M1**) [9]. Consequently, almost 90% of the dipyrid-2-ylamide ligands were not involved in complex formation, which is of importance for the catalytic behavior of the supported catalyst and for metal leaching. Poly(**M1**-*b*-**M2**)**-Rh** was again subject to cmc measurements. A value of 2.2 × 10^−6 ^mol L^−1^ was found, indicating that the loading with Rh(I) hardly changes the cmc value (Figure S4, Supporting Information File 1).

### Hydroformylation under micellar conditions

Generally, micellar catalysis [3,5,24–28] and in particular micellar set-ups, where catalysts covalently bound to amphiphilic polymers are used [2,4,29–33], have been reported to present suitable catalysts for numerous catalytic reactions. The hydroformylation under micellar conditions using catalysts bound to amphiphilic block copolymers was first reported by Nuyken et al. [34]. Using a poly(2-oxazoline)-based amphiphilic copolymer and a RhBr(1,3-dialkylimidazol-2-ylidene-based catalyst, selectivities (*n*:*iso*) of ~3 at 40% conversion and an activity (TOF_0_) of 1630 h^−1^ in the hydroformylation of 1-octene was observed [35]. Here, we used poly(**M1**-*b*-**M2**)**-Rh** for the hydroformylation of 1-octene in water. For purposes of comparison, the results obtained were compared to those previously obtained with the homogeneous analogue CH_3_CON(Py)_2_RhCl(COD) (**C1**) [9].

As already described [9], the dipyridylamide-based Rh-catalyst **C1** is a very fast isomerization catalyst. Consequently, considerable amounts of *iso*-octanes and a low *n*:*iso* ratio of 0.9 was reported for this catalyst (Table 1, entry 3).

**Table 1:** Results for the hydroformylation of 1-octene^a^.

**No.**	**Catalyst**	**Solvent**	**TON^c^**	**TOF_0_**	***n*:*iso***

1	poly(**M1**-*b*-**M2**)-**Rh**	water	3800	1200	1.5
2	poly(**M1**-*b*-**M2**)-**Rh**^b^	water	4400	1200	2.3
3	**C1**	toluene	4500	2700	0.9
4	**C1**^b^	toluene	4700	1600	1.6

^a^catalyst:substrate ratio = 1:5000, *t* = 4 h, *T* = 70 °C. ^b^triphenylphosphite:substrate = 10:5000. ^c^based on the aldehydes formed.

**Figure 1:**
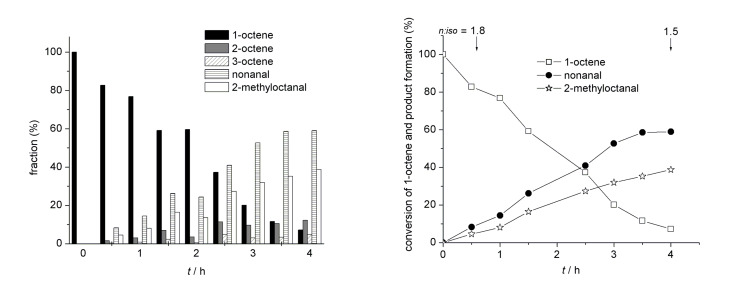
Conversion (%) of 1-octene, product formation, product distribution, as well as time dependant *n*:*iso* ratio in the hydroformylation of 1-octene in water in the presence of poly(**M1**-*b*-**M2**)-**Rh**.

**Figure 2:**
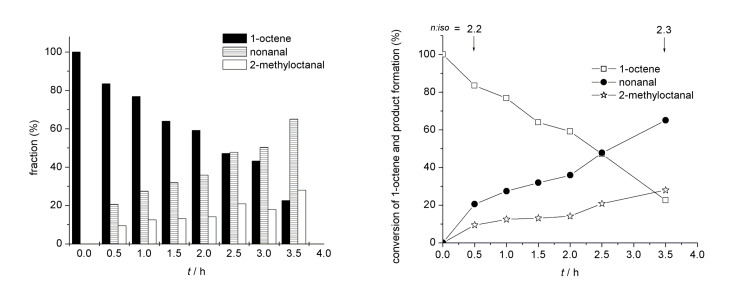
Conversion of 1-octene, product formation and product distribution in the hydroformylation in water in the presence of poly(**M1**-*b*-**M2**)-**Rh** and triphenylphosphite.

When poly(**M1**-*co*-**M2**)**-Rh** was used in water, a turn-over number (TON) of 4700 and an initial turn-over frequency (TOF_0_) of 1200 h^−1^ was observed. The *n*:*iso* ratio, however, was higher than the one obtained with **C1** in toluene, i.e. 1.5 vs. 0.9 for **C1** (Table 1, entry 1, Figure S5, Supporting Information File 1). At 40% conversion, an *n*:*iso* ratio of 1.6 (Figure S6, Supporting Information File 1) was found. A representative product distribution obtained with poly(**M1**-*co*-**M2**)**-Rh** is shown in Figure 1. This enhanced selectivity is attributed to the presence of a comparably large percentage (88%) of free dipyrid-2-ylamide ligands as well as to the high concentration of educts within the micelle. Both apparently suppress β-elimination in the alkyl-metal species. Consequently, a further addition of free ligand (e.g., triphenylphosphite), which is known to favor the formation of *n*-aldehydes in homogenous catalysis [10,36], the *n*:*iso* value could be further increased to 2.3 (Table 1, entry 2, Figure 2) and to 2.5 at 40% conversion, respectively (Figure S6, Supporting Information File 1). As a matter of fact, neither ethylheptanal or propylhexanal nor the parent 3- and 4-octenes was observed in this experiment.

By contrast, the *n*:*iso* value increased only to 1.6 with **C1** in toluene upon addition of excess ligand (Figure S7, Supporting Information File 1), clearly demonstrating the effect of the micelle, where both the reactants and the additional ligand (i.e. triphenylphosphite) accumulate. Finally and importantly, the leaching of Rh from poly(**M1**-*co*-**M2**)**-Rh** into the products was very low, resulting in metal contaminations of around 9 ppm. Again, the large excess of free dipyrid-2-ylamine ligand is thought to be responsible for this finding. Moreover, the polymer-bound catalyst can be recycled by extracting the products with diethyl ether and reused with only a minor change in activity. Thus, the TONs obtained were 4300 and 3900 for the first and second run.

## Conclusion

An amphiphilic block copolymer bearing a chelating *N,N*-dipyrid-2-ylamide-based ligand was prepared via ROMP using a Mo-based Schrock initiator. Loading with Rh(I) yielded a polymer-bound catalyst that was used for the hydroformylation of 1-octene. From the hydroformylation data obtained with the polymer-bound catalyst as well as with the model catalyst, it becomes clear that the use of a micellar catalyst favors the formation of the *n*-aldehyde by suppressing the isomerization propensity of a catalyst. Apparently, the higher concentration of the starting alkene inside the micelle effectively prevents β-elimination, an effect that can be further enhanced by adding free ligand that again accumulates inside the micelle. Further advantages in favor of a micellar setup are the low metal contamination of the products as well as the possibility of reuse.

## Experimental

All manipulations were performed under a N_2_ atmosphere in a glove box (LabMaster 130, MBraun, Garching, Germany) or by standard Schlenk techniques unless stated otherwise. Purchased starting materials were used without any further purification. Pentane, diethyl ether, toluene, CH_2_Cl_2_ and tetrahydrofuran (THF) were dried using a solvent purification system (SPS, MBraun). Benzene, *n*-hexane and dimethoxyethane (DME) were dried and distilled from sodium/benzophenone ketyl under argon. NMR spectra were recorded at room temperature on a Bruker AM 400 (400 MHz for proton and 100.6 MHz for carbon) and on a Bruker Avence 600 II**^+^** (600.25 MHz for proton and 150.93 MHz for carbon) spectrometer, respectively, unless specified otherwise. Proton and carbon spectra were referenced to the internal solvent resonance and are reported in ppm. Molecular weights and polydispersity indices (PDIs) of the polymers were determined by GPC at 40 °C on Waters columns (Styragel HR 4 DMF, 4.6 × 300 mm) in DMF vs. poly(styrene) using a Waters 717 plus autosampler and a Waters 2414 refractive index detector. For calibration, poly(styrene) samples (PDI < 1.02) with molecular weights within the range 162 < *M*_n_ <5,500,000 g/mol were used. The flow rate was 1.0 mL/min. Fluorescence testing was performed using a Perkin-Elmer luminescence spectrometer LS50B. IR spectra were recorded on a Bruker Vector 22 using ATR technology. Elemental analysis was carried out on Elementar Varia El (Analytik Jena). GC-MS investigations were carried out on a Shimadzu GCMS-QP5050 with an AOC-20i Autosampler using a SPB fused silica (Rxi-5MS) column (30 m × 0.25 mm × 0.25 μm film thickness, 60.6 kPa, temperature program: 70 °C – 300 °C, 25 min). The Schrock initiator Mo(*N*-2,6-Me_2_-C_6_H_3_)[CHC(CH_3_)_2_Ph][OCMe(CF_3_)_2_]_2_ **[Mo]** [22], *N,N-*dipyridyl-*endo*-norborn-5-ene-2-carbamide (**M1**) [9,37] and *N*-acetyl-*N,N*-dipyrid-2-yl (cyclooctadiene) rhodium chloride (**C1**) [9] were synthesized according to the literature.

***exo,exo*-[2-(3-Ethoxycarbonyl-7-oxabicyclo[2.2.1]hept-5-en-2-carbonyloxy)ethyl]trimethylammonium iodide (M2) *exo,exo-*7-Oxabicyclo[2.2.1]hept-5-ene-2,3-dicarboxylic acid mono(2-dimethylaminoethyl) ester (1):** *exo*-3,6-Epoxy-1,2,3,6-tetrahydrophtalic anhydride (10.0 g, 60.0 mmol), 2-dimethylaminoethanol (6.3 mL, 62.0 mmol, 1.03 equiv) and *p*-toluenesulfonic acid (590 mg, 3.1 mmol, 5 mol%) were dissolved in 120 mL of THF and stirred at room temperature for 8 h. The white precipitate was filtered, washed with diethyl ether and dried to afford a white solid; yield: 13.8 g (90.0%). ^1^H NMR (CDCl_3_): δ 2.70 (s, 6H), 2.78–2.81 (m, 2H), 3.01 (ddd, 1H, ^2^*J* = 13.7 Hz, *J* = 6.8 Hz, *J* = 2.8 Hz), 3.12 (ddd, 1H, ^2^*J* = 13.7 Hz, *J* = 7.3 Hz, *J* = 2.8 Hz), 4.37 (ddd, 1H, ^2^*J* = 13.3 Hz, *J* = 7.3 Hz, *J* = 2.8 Hz), 4.51 (ddd, 1H, ^2^*J* = 13.3 Hz, *J* = 6.8 Hz, *J* = 2.8 Hz), 5.24 (s br, 2H), 6.37 (dd, 1H, *J* = 5.7 Hz, *J* = 1.6 Hz), 6.47 (dd, 1H, *J* = 5.7 Hz, *J* = 1.6 Hz).

***exo,exo*-7-Oxabicyclo[2.2.1]hept-5-ene-2,3-dicarboxylic acid 2-(2-dimethylaminoethyl) ester 3-ethyl ester (2):** **1** (1.0 g, 3.9 mmol) was dissolved in 20 mL of EtOH (*c* = 0.2 M). SOCl_2_ (0.4 mL, 5.9 mmol, 1.5 equiv) was added dropwise at room temperature and the reaction mixture was stirred at room temperature for 8 h. The solvent was removed in vacuo, the green residue dissolved in 25 mL of CH_2_Cl_2_ and washed with 10 mL of sat. NaHCO_3_ solution. The aqueous phase was extracted with CH_2_Cl_2_ (3 × 10 mL) and the combined organic layers were dried over MgSO_4_. Finally, the solvent was removed under reduced pressure to afford a yellow oil; yield: 695 mg (60%). ^1^H NMR (CDCl_3_): δ 1.29 (t, 3H, *J* = 7.2 Hz), 2.30 (s, 6H), 2.59 (t, 2H, *J* = 5.8 Hz), 2.81 (d, 1H, *J* = 9.0 Hz), 2.88 (d, 1H, *J* = 9.0 Hz), 4.16–4.21 (m, 3H), 4.23–4.28 (m, 1H), 5.28 (s, 1H), 5.29 (s, 1H), 6.47 (s, 2H).

***exo,exo*-[2-(3-Ethoxycarbonyl-7-oxabicyclo[2.2.1]hept-5-ene-2-carbonyloxy)ethyl]trimethylammonium iodide (M2):** **2** (659 mg, 2.3 mmol) was dissolved in 2 mL of CH_3_I at 0 °C. The reaction mixture was stirred for 30 min at 0 °C and for 8 h at room temperature. The resulting yellow precipitate was isolated by filtration, dried and recrystallized from boiling ethanol. The crude product was dissolved in CH_2_Cl_2_ and precipitated with hexane to afford a white solid; yield: 875 mg (88%). ^1^H NMR (CDCl_3_): δ 1.30 (t, 3H, *J* = 7.2 Hz), 2.86 (d, 1H, ^2^*J* = 9.0 Hz), 2.94 (d, 1H, ^2^*J* = 9.0 Hz), 3.54 (s, 9H), 4.16 (q, 2H, *J* = 7.2 Hz), 4.10–4.23 (m, 2H), 4.56 (dd, 1H, ^2^*J* = 14.0 Hz, *J* = 5.1 Hz), 4.74 (dd, 1H, ^2^*J* = 14.0 Hz, *J* = 6.9 Hz), 5.15 (s br, 1H), 5.43 (s br, 1H), 6.48 (dd, 1H, *J* = 5.8 Hz, J = 1.4 Hz), 6.50 (dd, 1H, *J* = 5.8 Hz, *J* = 1.6 Hz). ^13^C NMR (CDCl_3_, 100 MHz): δ 14.24 (CH_3_), 46.77 (CH), 47.91 (CH), 54.88 (CH_3_), 58.65 (CH_2_), 61.69 (CH_2_), 64.75 (CH_2_), 80.10 (CH), 80.87 (CH), 136.52 (CH), 136.76 (CH), 171.19 (C_q_), 172.00 (C_q_). IR (ATR) ν (cm^−1^) = 3433 (w), 3002 (w), 2977 (w), 1732 (ss), 1190 (ss), 1157 (ss) 913 (s), 887 (s). Elemental analysis**:** calcd. for C_15_H_24_INO_5_: C, 42.36; H, 5.69; N, 3.29; found: C, 42.25; H, 5.71; N, 3.29.

**Homopolymerization of M1: M1** (100 mg, 0.339 mmol) was dissolved in 5 mL of CH_2_Cl_2_ and treated with a solution of **[Mo]** (2.4 mg, 0.0034 mmol) in 1 mL of CH_2_Cl_2_. The yellow reaction mixture was stirred for 2 h at room temperature. A solution of ferrocene carboxaldehyde (10-fold excess based on initiator) in CH_2_Cl_2_ was added and the mixture was stirred for 1 h at room temperature. The polymer was precipitated by addition of pentane, filtered off, thoroughly washed with pentane and dried; yield: 85 mg (85%). Poly(**M1**): *M*_n(calc)_ = 29400 g mol^−1^, *M*_n(found)_ = 37900 g mol^−1^; PDI = 1.21. ^1^H NMR (CDCl_3_): δ 8.39 (broad d, 2H Py), 7.72 (broad d, 2H Py), 7.34 (broad d, 2H Py), 7.14 (broad d, 2H Py), 5.72–5.43 (broad t, 2H -CH=CH-), 3.12 (broad s, 1H NBE), 2.89–2.23 (broad d, 2H NBE), 2.20–1.20 (broad d, 4H NBE). ^13^C NMR (CDCl_3_): δ 175.0 (C=O), 154.8, 148.9, 137.8, 134.6 and 130.2 (all Py), 122.6 and 121.9 (-CH=CH-), 53.3, 47.6, 41.9 and 37.3 (all NBE). FT-IR (ATR mode): ν (cm^−1^) **=** 2995 (w), 1672 (s), 1577 (s), 1426 (m), 1239 (w), 1048 (m), 739 (m).

**Homopolymerization of M2: M2** (100 mg, 0.235 mmol) was dissolved in 5 mL of CH_2_Cl_2_ and treated with a solution of **[Mo]** (3.0 mg, 0.0042 mmol) in 1 mL of CH_2_Cl_2_. The resulting white cloudy reaction mixture was stirred for 2 h at room temperature. A solution of ferrocene carboxaldehyde (10-fold excess based on initiator) in CH_2_Cl_2_ was added and the mixture was then stirred for 1 h at room temperature. The polymer was filtered off, thoroughly washed with pentane and dried; yield: 90 mg (90%). Poly(**M2**): *M*_n(calc)_ = 23600 g mol^−1^, *M*_n(found)_ = 24500 g mol^−1^, PDI = 1.36. ^1^H NMR (CDCl_3_): δ 5.83 (broad s, 2H -CH=CH-), 5.17 (broad s, 2H CH_2_), 4.70 (broad s, 2H CH_2_), 4.21 (broad d, 4H CH_2_), 3.51 (broad s, 13 H NBE + NCH_3_), 1.26 (broad s, 3H CH_3_). ^13^C NMR (CDCl_3_): δ 171.3 and 170.4 (C=O), 132.7(-CH=CH-), 77.2 (NBE), 64.6, 61.8, 59.4 (CH_2_), 55.1, 53.8 and 52.9 (NCH_3_), 14.1 (CH_3_). FT-IR (ATR mode): ν (cm^−1^) **=** 3428 (w), 2969 (w), 1726 (s), 1427 (m), 1181 (w), 1018 (m), 953 (m).

**Synthesis of poly(M1-*b*-M2):** A solution of [**Mo**] (2.4 mg, 0.0034 mmol) in 1 mL of CH_2_Cl_2_ was added to one of **M1** (30.0 mg, 0.102 mmol) in 1 mL of CH_2_Cl_2_. The reaction mixture was stirred for 20 min and a small amount was withdrawn for GPC measurements. **M2** (100 mg, 0.235 mmol) was dissolved in 2 mL of CH_2_Cl_2_ and this solution was added to the reaction mixture. The resulting white cloudy reaction mixture was stirred for another 90 min. The polymerization was terminated with ferrocene carboxaldehyde (10-fold excess based on initiator) and the reaction was stirred for a further hour. The polymer was filtered off, thoroughly washed with pentane and dried; yield: 100 mg (76%). *M*_n_ = 44100 g mol^−1^, *M*_w_ = 61100 g mol^−1^, PDI = 1.38. ^1^H NMR (CDCl_3_): δ 8.40, 7.92, 7.43, 7.33 (broad s, Py), 5.79 (broad s, -CH=CH-), 5.17 (broad s, CH_2_), 4.70 (broad s, CH_2_), 4.21 (broad d, CH_2_), 3.51 (broad s, NBE + NCH_3_), 1.87 (broad, NHE), 1.27 (CH_3_). ^13^C NMR (CDCl_3_): δ 175.6 (C=O), 171.3 and 170.4 (C=O), 154.8, 148.9, 137.8, 134.6 and 130.2 (all Py), 122.8 and 122.2 (-CH=CH-), 77.0 (NBE), 64.6, 61.6, 59.3 (CH_2_), 52.8 (NCH_3_), 22.3, 14.1 (CH_3_). FT-IR (ATR mode): ν (cm^−1^) **=** 3404 (w), 2937 (w), 1727 (s), 1679 (m), 1586 (m), 1467 (m), 1432 (w), 1375 (m), 1238 (w), 1184 (s), 1097 (w), 1018 (m), 954 (m), 749 (m).

**Synthesis of poly(M1-*b*-M2)-Rh:** [Rh(COD)_2_Cl]_2_ (8 mg) was dissolved in CH_2_Cl_2_. This solution was added dropwise to a suspension of poly(**M1**-*b*-**M2**) (100 mg) in 5 mL of CH_2_Cl_2_. The mixture was stirred for 10 h at room temperature; then the polymer was filtered off, thoroughly washed with pentane and dried; yield: 100 mg (92%). The Rh-content was determined by ICP-OES: 18 mg Rh/g copolymer (12% of the theoretical loading).

**cmc determination of poly(M1-*b*-M2) and poly(M1-*b*-M2)-Rh:** The cmc in water were determined by fluorescence spectroscopy [23] using 6-*p*-toluidene-2-naphthylsufonic acid (10^−6^ M) as the fluorescence probe. Polymer solutions were prepared in a concentration range from 1 × 10^−4^–1 × 10^–13^ mol L^–1^. cmc of poly(**M1**-*b*-**M2**) = 2.8 × 10^−6^ mol L^−1^; cmc of poly(**M1**-*b*-**M2**)-**Rh** = 2.2 × 10^−6^ mol L^−1^.

**Quantification of Rh:** The amount of Rh bound to the block copolymers or leached into the products was determined by dissolving a 20.0 mg amount of the polymer or the product mixture in aqua regia (25 mL). Rh was quantified by ICP-OES at λ = 343.489 nm, measuring the background at λ_1_ = 343.410 and λ_2_ = 343.600 nm, respectively. A 1000 ppm Rh standard (1N HNO_3_, Merck, Germany) was used to prepare standards of 0, 0.100, 1.00 and 10.00 ppm.

**General procedure for hydroformylation:** The reaction was carried out in a 300 mL Parr high-pressure reactor. The reactor was evacuated, flushed with argon and filled with the catalyst, water or toluene (30 mL), and 1-octene (1.0 g, 0.0090 mol), leading to a substrate to catalyst ratio of 5000:1. *tert*-Butylbenzene (1.00 mL) was added as internal standard. To purge the reactants, the mixture was pressurized with a 1:1 mixture of CO and H_2_ up to a pressure of 30 bar and then the pressure was released. Finally, the pressure was adjusted to 50 bar with the aid of a back pressure regulator. The autoclave was heated to 70 °C and kept at this temperature. Samples were taken every 30 min. The isomeric 1-octenes and aldehydes were identified and quantified by their MS spectra as well as by their retention times using commercially available compounds as reference material.

**Recyclability studies with poly(M1-*b*-M2)-Rh:** Hydroformylations were carried out as described above. For recycling, the product was extracted with diethyl ether (4 × 10 mL). Fresh 1-octene (1.0 g, 0.0090 mol), and *tert*-butylbenzene (1.0 mL) were added to the aqueous, catalyst-containing phase and the reaction was run under the same conditions as before. The TONs in two consecutive cycles as determined by GC-MS were 4300 and 3900, respectively.

## Supporting Information

Supporting Information contains polymerization kinetics of **M1** by the action of Mo(*N*-2,6-Me_2_ C_6_H_3_)(CHCMe_2_Ph)(OCMe(CF_3_)_2_)_2_, GPC-traces (DMF) of poly(**M1**) and poly(**M1**-*b*-**M2**); cmc measurements for poly(**M1**-*b*-**M2**) and poly(**M1**-*b*-**M2**)-**Rh** in water; conversion of 1-octene, product formation, product distribution in the hydroformylation of 1-octene in toluene in the presence of **C1,** **C1** and triphenylphosphite; *n*:*iso* selectivities for catalysts poly(**M1**-*b*-**M2**)-**Rh**) and poly(**M1**-*b*-**M2**)-**Rh** with triphenylphosphite as a function of conversion.

File 1 Graphical representations of measurements.

## References

[1] Kotre T, Zarka M T, Krause J O, Buchmeiser M R, Weberskirch R, Nuyken O (2004). Macromol Symp.

[2] Nuyken O, Persigehl P, Weberskirch R (2002). Macromol Symp.

[3] Nuyken O, Weberskirch R, Kotre T, Schoenfelder D, Wörndle A, Buchmeiser M R (2003). Polymers for micellar catalysis. Polymeric Materials in Organic Synthesis and Catalysis.

[4] Nuyken O, Weberskirch R, Bortenschlager M, Schönfelder D (2004). Macromol Symp.

[5] Oehme G, Cornils B, Herrmann W A, Horvath I T (2005). Micellar Systems. Multiphase Homogeneous Catalysis.

[6] Pawar G, Bantu B, Weckesser J, Blechert S, Wurst K, Buchmeiser M R (2009). Dalton Trans.

[7] Weberskirch R, Preuschen J, Spiess H W, Nuyken O (2000). Macromol Chem Phys.

[8] Krause J O, Zarka M T, Anders U, Weberskirch R, Nuyken O, Buchmeiser M R (2003). Angew Chem.

[9] Bantu B, Wurst K, Buchmeiser M R (2007). J Organomet Chem.

[10] Ungváry F (2007). Coord Chem Rev.

[11] Holmberg K (2007). Eur J Org Chem.

[12] Buchmeiser M R, Dubois P, Coulembier O, Raquez J-M (2009). Ring-Opening Metathesis Polymerization. Handbook of Ring-Opening Polymerization.

[13] Buchmeiser M R (2000). Chem Rev.

[14] Stubenrauch K, Moitzi C, Fritz G, Glatter O, Trimmel G, Stelzer F (2006). Macromolecules.

[15] Buchmeiser M R, Wurst K (1999). J Am Chem Soc.

[16] Buchmeiser M R, Lubbad S, Mayr M, Wurst K (2003). Inorg Chim Acta.

[17] Trnka T M, Grubbs R H (2001). Acc Chem Res.

[18] Grubbs R H (2006). Angew Chem.

[19] Schrock R R (1995). Polyhedron.

[20] Schrock R R, Grubbs R H (2003). The Discovery and Development of High-Oxidation State Mo and W Imido Alkylidene Complexes for Alkene Metathesis. Handbook of Metathesis.

[21] Schrock R R (2005). Chem Commun.

[22] Oskam J H, Fox H H, Yap K B, McConville D H, O’Dell R, Lichtenstein B J, Schrock R R (1993). J Organomet Chem.

[23] Ananthapadmanabhan K P, Goddard E D, Turro N J, Kuo P L (1985). Langmuir.

[24] Dwars T, Haberland J, Grassert I, Oehme G, Kragl U (2001). J Mol Catal A: Chem.

[25] Morawetz H (1969). Adv Catal.

[26] Lipshutz B H, Aguinaldo G T, Ghorai S, Voigtritter K (2008). Org Lett.

[27] Mingotaud A-F, Mingotaud C, Moussa W (2008). J Polym Sci, Part A: Polym Chem.

[28] Oheme G, Grassert I, Paetzold E, Meisel R, Drexler K, Fuhrmann H (1999). Coord Chem Rev.

[29] Kotre T, Nuyken O, Weberskirch R (2002). Macromol Rapid Commun.

[30] Zarka T M, Nuyken O, Weberskirch R (2003). Chem–Eur J.

[31] Kotre T, Nuyken O, Weberskirch R (2004). Macromol Chem Phys.

[32] Schönfelder D, Nuyken O, Weberskirch R (2005). J Organomet Chem.

[33] Schönfelder D, Fischer K, Schmidt M, Nuyken O, Weberskirch R (2005). Macromolecules.

[34] Zarka M T, Bortenschlager M, Wurst K, Nuyken O, Weberskirch R (2004). Organometallics.

[35] Bortenschlager M, Wittmann A, Schoellhorn N, Weberskirch R, Nuyken O (2005). Polym Prepr.

[36] Weissermel K, Arpe H J (1993). Industrial Organic Chemistry.

[37] Sinner F, Buchmeiser M R, Tessadri R, Mupa M, Wurst K, Bonn G K (1998). J Am Chem Soc.

