# Supplementary material for: Ring opening metathesis polymerization-derived block copolymers bearing chelating ligands: synthesis, metal immobilization and use in hydroformylation under micellar conditions
Source: Beilstein J Org Chem. 2010 Mar 23;6:28. doi: 10.3762/bjoc.6.28 (PMC2874313; doi:10.3762/bjoc.6.28)
Supplement: File 1 — Graphical representations of measurements. [file Beilstein_J_Org_Chem-06-28-s001.pdf]

Supporting Information

to

Ring opening metathesis polymerization-derived block copolymers bearing chelating ligands: synthesis, metal immobilization and use in hydroformylation under micellar conditions

Gajanan M. Pawar[1], Jochen Weckesser[2], Siegfried Blechert[*2] and Michael R. Buchmeiser[*1]

[1]Lehrstuhl für Makromolekulare Stoffe und Faserchemie, Institut für Polymerchemie, Universität Stuttgart, Pfaffenwaldring 55, D-70550 Stuttgart, Germany and [2]Institut für Chemie, Technische Universität Berlin, Straße des 17. Juni 135, D-10623 Berlin, Germany

Email: Michael R. Buchmeiser - michael.buchmeiser@ipoc.uni-stuttgart.de; Tel.: +49 (0)711-685-64075; Fax: +49 (0)711-685-64050;

Siegfried Blechert - blechert@chemie.tu-berlin.de

*Corresponding author

Graphical representations of measurements

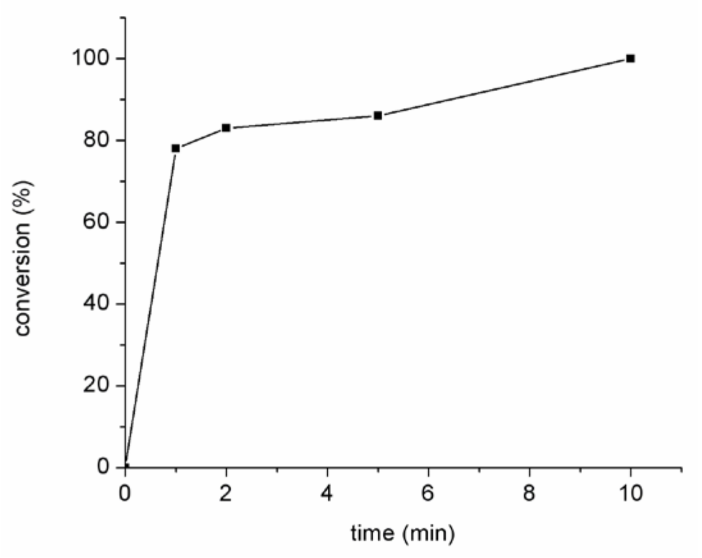

Figure S1: Polymerization kinetics of **M1** by the action of Mo(*N*-2,6-Me_2 C_6H_3)($CHCMe_2Ph$)($OCMe(CF_3)_2)_2$.

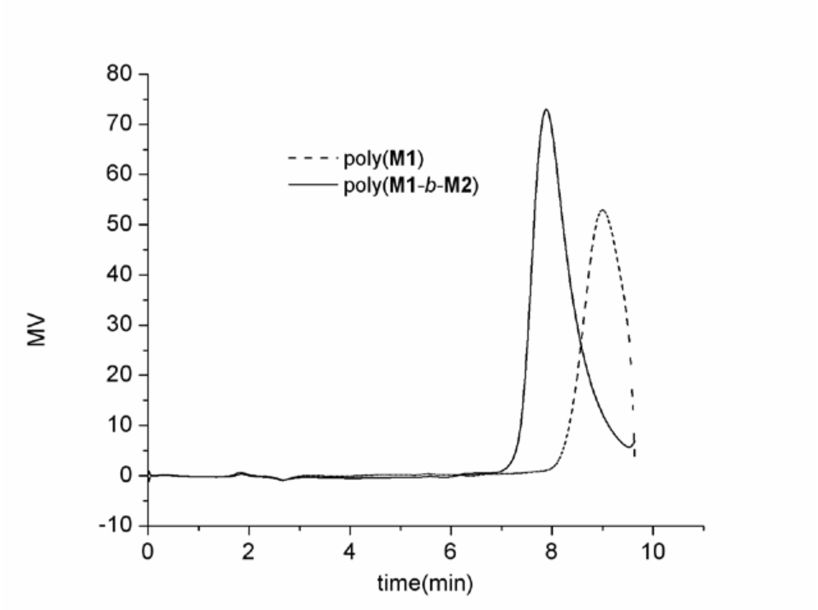

Figure S2: GPC-traces (DMF) of poly(**M1**) and poly(**M1**-*b*-**M2**) prepared there from.

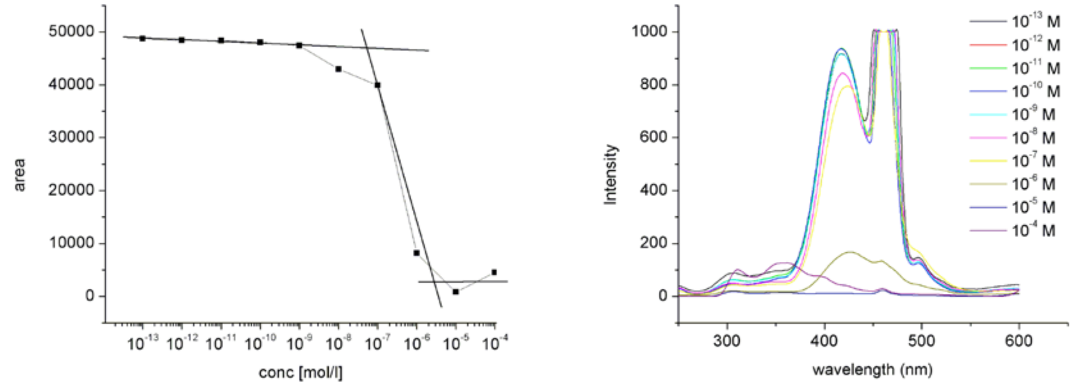

Figure S3: cmc measurements for poly(**M1**-*b*-**M2**) in water.

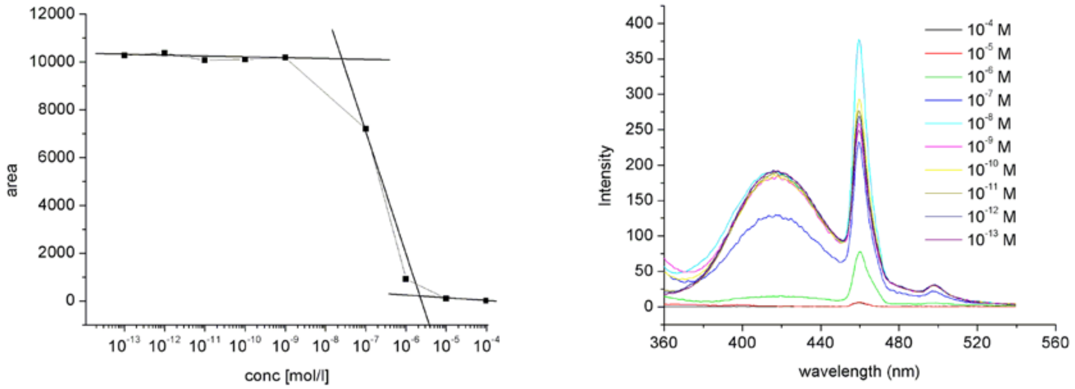

Figure S4: cmc measurements for poly(**M1**-*b*-**M2**)-**Rh** in water.

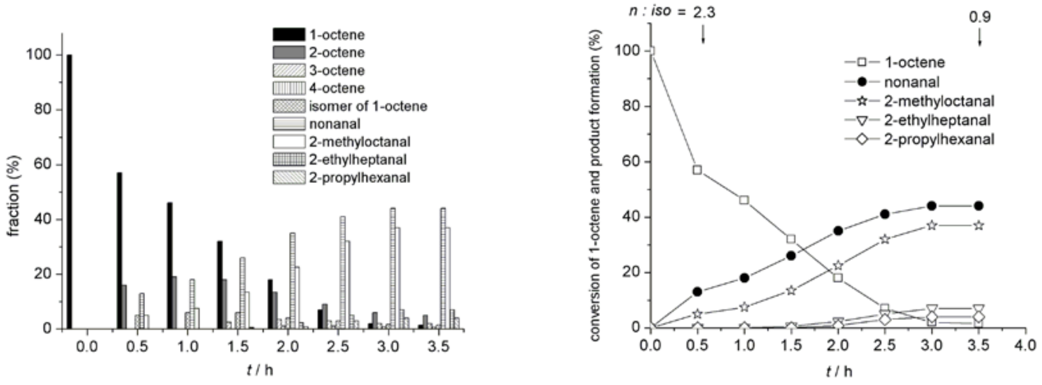

Figure S5: Conversion of 1-octene, product formation, product distribution in the hydroformylation of 1-octene in toluene in the presence of **C1**[2].

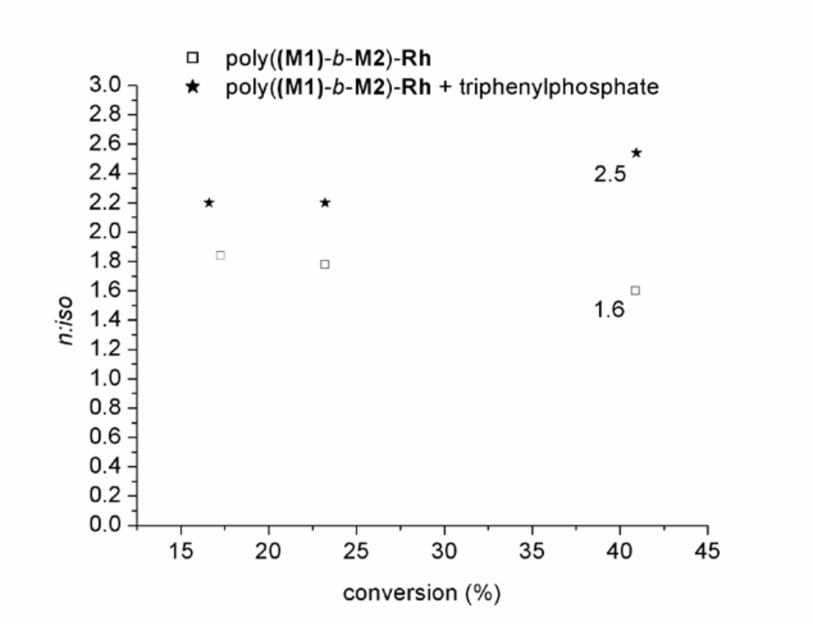

Figure S6: *n:iso* Selectivities for catalysts poly(**M1**-*b*-**M2**)-**Rh** (□) and poly(**M1**-*b*-**M2**)-**Rh** (★) with triphenylphosphite as a function of conversion.

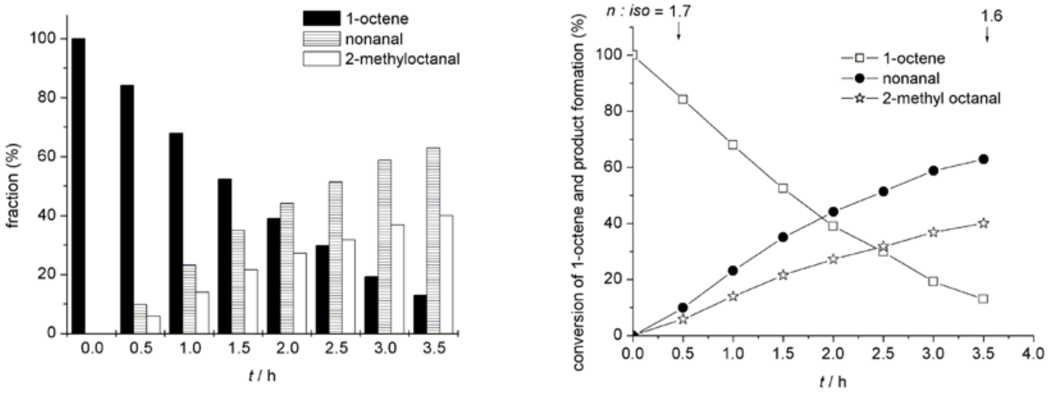

Figure S7: Conversion of 1-octene, product formation, product distribution in the hydroformylation of 1-octene in toluene in the presence of **C1** and triphenylphosphite.
